# Satisfaction with care after total hip or knee replacement predicts self-perceived health status after surgery

**DOI:** 10.1186/1471-2474-10-150

**Published:** 2009-12-03

**Authors:** Cédric Baumann, Anne Christine Rat, Georges Osnowycz, Didier Mainard, Christian Cuny, Francis Guillemin

**Affiliations:** 1 Nancy-Université, Université Paul Verlaine Metz, Université Paris Descartes, EA 4360 Apemac, Nancy, France; 2 INSERM, CIC-EC CIE6, Nancy, France; 3 CHU Nancy, Epidémiologie et Evaluation Cliniques, Nancy, France; 4 CHG Neufchateau, Chirurgie B, Neufchateau, France; 5 CHU Nancy, Chirurgie orthopédique et traumatologique, Nancy, France; 6 CHR Metz-Thionville, Chirurgie orthopédique et traumatologique, Metz, France

## Abstract

**Background:**

Inpatient satisfaction with care is a standard indicator of the quality of care delivered during hospitalization. Total hip and knee replacement (THR/TKR) for osteoarthritis (OA) are among the most successful orthopaedic interventions having a positive impact on health-related quality of life (HRQoL). The aim was to evaluate the effect of satisfaction shortly after hospital discharge on 1-month, 6-month and 1-year Medical Outcomes Study 36-item Short Form (SF-36) scores for OA patients after THR and TKR, controlling for patient characteristics, clinical presentation and preoperative SF-36 scores.

**Methods:**

A multicenter prospective cohort study recruited 231 patients with OA scheduled to receive THR or TKR. Satisfaction was assessed by the Patients Judgment of Hospital Quality (PJHQ) questionnaire and HRQoL by the SF-36 questionnaire. Linear models for repeated measures assessed the relation between satisfaction (scores were dichotomized) and postoperative SF-36 scores.

**Results:**

Of 231 participants, 189 were followed up 12 months after discharge (mean age 69 SD = 8; 42.6% male). The mean length of hospital stay was 13.5 (SD = 4) days. After adjustment for preoperative SF-36 scores, sociodemographic and clinical patient characteristics, satisfied patients (PJHQ score > 70) had higher SF-36 scores 1 year after surgery than did less-satisfied patients. Admission, medical care, and nursing and daily care scores mainly predicted bodily pain, mental health, social functioning, vitality and general health scores of the SF-36.

**Conclusion:**

Besides being a quality-of-care indicator, immediate postoperative patient satisfaction with care may bring a new insight into clinical practice, as a predictor of self-perceived health status after surgery.

## Background

For patients with severe osteoarthritis (OA), one of the primary goals of total hip or knee replacement (THR/TKR) is to maximize function in everyday life and to reach a higher level of well-being [1]. THR and TKR are among the most effective orthopaedic interventions [2] and substantially improve postoperative health-related quality of life (HRQoL) 3 to 6 months after surgery [3-8].

The most frequently used tool to assess HRQoL is the Medical Outcomes Study 36-item Short Form (SF-36), a generic self-administered questionnaire. Many predictors of SF-36 scores have been investigated [5,9,10], the most common being preoperative SF-36 scores, which are positively correlated with improvement in SF-36 scores after surgery [11,12]. Some studies have suggested medical care characteristics (medication, type of prosthesis), physician-related characteristics (sex, experience) [13] and effective patient-physician communication [14] as potential predictors of improvement in SF-36 score. Indeed, if health problems and personal factors are major HRQoL determinants, environmental factors, as defined by The International Classification of Functioning, Disability and Health, are also major determinants. For patients undergoing THR or TKR, a better knowledge of these environmental factors could help improve care with specific interventions. Health system characteristics (hospital volume, center), could also have an important impact on outcome. Since HRQoL of patients undergoing surgery is related to not only objective clinical facts but also environmental factors, especially medical care and patient satisfaction with care, knowing whether and to what extent patients' experiences in the hospital influence their health status after arthroplasty would be of interest. We could assess the impact of a program of care or, more generally, a process of care, and further identify new factors to improve OA patients' HRQoL after surgery.

Usually, patient satisfaction is treated as an endpoint to assess health care organisation. Knowledge of factors associated with inpatient satisfaction helps target patient groups at risk of dissatisfaction with the process of care [15,16]. Hospital leaders need information about opportunities for improving factors under their control that contribute to health status improvement. Satisfaction with care also has some impact on further health and health care use. Indeed, patients satisfied with care are more prone to comply with treatment regimens and physician advice [17,18] and to continue to use medical care services [19,20]. Identifying such satisfaction dimensions with any health care process would help improve knowledge about the efficacy of the inpatient health care modalities and increase hospital effectiveness.

The aim of this study was to evaluate the effect of satisfaction with care, measured shortly after hospital discharge, on 1-year SF-36 scores for OA patients after THR and TKR, controlling for sociodemographic characteristics, clinical presentation and preoperative SF-36 scores.

## MethodS

### Design

We conducted a multicenter prospective cohort study of patients with hip or knee OA scheduled to receive THR or TKR. We prospectively followed patients from their referral before THR or TKR to 1 year after discharge from hospital.

The study protocol was approved by the Regional Ethics Committee and the "Comité National Informatique et Liberté" (CNIL n°02-1181), which ensures the confidentiality of information. All patients gave their informed consent to participate in the study.

### Participants and Settings

Patients were enrolled between April 1, 2002, and June 30, 2004, at outpatient surgery clinics in 3 hospitals (Nancy, Metz and Neufchateau) in the Lorraine region, eastern France.

Inclusion criteria were hip or knee OA according to American College of Rheumatology criteria [21,22], age older than 18 years, indication for primary unilateral THR or TKR, speaking French and sufficient cognitive function to complete a self-administered questionnaire. Exclusion criteria were an indication for THR or TKR other than for OA and repeat THR or TKR within 1 year of a first operation.

To detect a true difference of 8 points in SF-36 scores between satisfied and less-satisfied patients 1 year after surgery, we used the minimal clinically important difference (MCID) of 5 to 10 points to evaluate differences in scores; the developers of the SF-36 consider this difference to be under the threshold of a meaningful difference. With a specified power of 80% and a type I error of 5%, a standard deviation of 10 and a ratio of satisfied to less-satisfied patients of 1, the sample size needed was 190 patients followed up 1 year after discharge from hospital. With 20% of patients potentially expected lost to follow-up, we sought to include 230 patients.

### Measures

Data were collected by surgeons before and after patient hospitalization (during the pre- and postoperative consultations) and after discharge at home (by self-administered questionnaire). A research assistant ensured quality of data collection.

*Before surgery*, sociodemographic data, clinical characteristics, including disease severity factors, were collected by medical interview, clinical examination, and a review of medical records; the SF-36 questionnaire was completed during the preoperative consultation.

*After surgery*, approximately 1 month after discharge, patients completed a mailed questionnaire (the Patient Judgements of Hospital Quality [PJHQ]) assessing their satisfaction with care during hospitalization. At 1, 6 and 12 months after discharge, just before consultation with surgeon, nursing staff gave each patient the SF-36 questionnaire to complete and return by mail. At 6 and 12 months after discharge, disease severity factors were assessed during patients' consultation with their surgeons.

#### Health-related quality of life

The SF-36 has been translated and adapted into French [23]. The survey measures HRQoL in 8 dimensions: physical functioning, physical role, bodily pain, mental health, emotional role, social functioning, vitality, and general health. All dimension scores represent the mean of item values obtained when the number of missing values is no more than half of the total; otherwise, the score is declared missing. Dimension scores were standardized from 0 to 100 (best HRQoL).

#### Satisfaction with care

The PJHQ questionnaire assessing inpatient satisfaction with care[24] has been translated and adapted into French [16]. This scale contains 34 questions covering 5 specific hospital practices: admission (4 items), nursing and daily care (8 items), medical care (4 items), information (6 items), and hospital environment and ancillary staff (12 items). Each item is rated on a 5-point Likert scale (poor, fair, good, very good, excellent). All dimension scores represent the mean of item values obtained when the number of missing values is no more than half of the total; otherwise, the score is declared missing. Scores were standardized from 0 to 100 (greatest satisfaction).

#### Other measures

Sociodemographic characteristics, including sex, age, family situation (single/married), residence (rural/urban), years of schooling (< 6, 6-12, > 12), occupational activity (paid work/not working), and retirement (yes/no), were collected at inclusion, during the preoperative consultation. The clinical parameters included diagnosis, body mass index (BMI), and Charlson Comorbidity Index score [25]. Disease severity was assessed before and after surgery by pain score (visual analog scale [VAS], 0-100 mm), walking distance (i.e., maximal walking distance [in meters] without stopping reported by the patient to the surgeon at the clinical examination during the preoperative consultation), Harris score (for hip OA) [26] and Index of Severity for Knee score (ISK, disease score for knee OA) [27]. Kellgren staging of X-rays [28] was assessed before surgery. We also collected data on perioperative and postoperative complications, the ability to choose a hospital (yes/no), waiting time to surgery (days), hospital stay (days), ambulatory rehabilitation after discharge (yes/no) and whether the patient was treated at an inpatient rehabilitation department (yes/no).

### Statistical analysis

#### Descriptive analysis

All variables were analyzed for the entire sample and separately for THR or TKR. Descriptive statistics are presented as means and standard deviations for continuous variables and as absolute and relative frequencies for categorical variables. We compared SF-36 scores and clinical parameters over 4 measurement times (1 preoperative and 3 postoperative) using ANOVA and the Bonferroni procedure for correction of multiple testing.

#### Bivariate analysis

For each dimension of the SF-36, we calculated the differences in scores between 1 and 6 months (Δ_6-1_) and 1 and 12 months (Δ_12-1_) after surgery. The association between patient characteristics and clinical parameters for both Δ_6-1 _and Δ_12-1 _HRQoL were analyzed by Student *t *test, ANOVA and Pearson correlation.

#### Multivariate analysis

Candidate variables with a significance level of 10% from bivariate analysis were entered in multivariate models. The impact of inpatient satisfaction with care on SF-36 scores over time after discharge was assessed by use of a linear model for repeated measures.

All dimensions of the SF-36 questionnaire were treated as dependent continuous variables. We dichotomized the PJHQ score for easier interpretation of the main results. The cut-off value of 70 corresponding most closely to the median satisfaction scores (table 1) allowed for obtaining 2 balanced groups and can be used in future studies. We used the terms "more satisfied" and "less satisfied" to qualify patients with scores > 70 and ≤ 70. This cut-off value is close to the value obtained from another study of inpatients discharged from 12 medical and surgical services specializing in cardiovascular, respiratory, urinary and locomotor system diseases at Nancy University Hospital Center [16]. Satisfaction scores as binary variables were adjusted for preoperative SF-36 scores, sex, age, site of joint replacement, center and variables selected from bivariate analysis. A time-effect interaction on postoperative SF-36 scores was considered in multivariate analysis when time and independent variables were simultaneously significant.

**Table 1:** Baseline Characteristics and Satisfaction Scores for Patients Undergoing Total Hip or Knee Replacement Surgery

		All patients n = 189				Total hip replacement n = 126				Total knee replacement n = 63			
				
		median [Q1-Q3]		n (%)		median [Q1-Q3]		n (%)		median [Q1-Q3]		n (%)	
**Age**		69	[65-75]			69	[64-75]			70	[65-75]		
**Sex**	**Male**			80	(42.6)			56	(44.8)			24	(38.1)
**Family situation**	**Spouse**			140	(77.3)			94	(78.3)			46	(75.4)
**Retired**	**Yes**			148	(92.5)			93	(92.1)			55	(93.2)
**Occupational activity**	**Yes**			25	(11.2)			24	(16.3)			1	(1.3)
**Residence**	**Rural (Urban)**			138	(75.8)			95	(78.5)			43	(69.4)
**Years of schooling**	**< 6**			142	(77.6)			93	(75.6)			49	(81.7)
	**6-12**			35	(19.1)			28	(22.8)			7	(11.7)
	**> 12**			6	(3.3)			2	(1.6)			4	(6.6)
**Body mass index**		28	[25-31]			28	[25-31]			29	[27-33]		
**Charlson comorbidity index**	**0**			97	(51.6)			64	(51.2)			33	(52.4)
	**1**			68	(36.2)			47	(37.6)			21	(33.3)
	**2 - 3**			23	(12.2)			14	(11.2)			9	(14.3)
**Kellgren stage at consultation**	**II**			31	(17.8)			17	(14.8)			14	(23.7)
	**III**			116	(66.7)			74	(64.3)			42	(71.2)
	**IV**			27	(15.5)			24	(20.9)			3	(5.1)
**Waiting time to surgery (days)**		49	[33-77]			47	[32-77]			54	[34-78]		
**Length-of-stay (days)**		13	[12-15]			13	[12-14]			13	[11-15]		
Satisfaction with care (PJHQ) scores*													
**Admission**		69	[50-81]			72	[50-81]			69	[56-87]		
**Medical care**		75	[58-87]			75	[67-87]			75	[58-94]		
**Nursing and daily care**		71	[56-84]			69	[56-84]			72	[56-81]		
**Hospital environment**		67	[55-80]			68	[55-82]			65	[55-77]		
**Information**		65	[50-80]			65	[50-80]			65	[50-80]		

[Q1-Q3] = [1st-3rd quartile]

* PJHQ = Patient Judgements of Hospital Quality; score from 0 (worse) to 100 (best satisfaction)

The level of type I error used to determine statistical significance in multivariate analysis was 5%. Data were recorded by use of Microsoft Access software. Statistical analysis involved use of SAS v8.02 (SAS Institute Inc., Gary, NC).

## Results

### Patient characteristics

Of 237 outpatients eligible at outpatient clinics, 6 did not undergo surgery. For the remaining 231 patients, SF-36 data were complete for 204 patients 1 month after discharge, for 196 at 6 months and for 189 patients at 12 months. The characteristics of the 189 patients at 12 months (126 THR, 63 TKR) is in Table 1. The mean age was (69 ± 8 years) and 42.6% were male. The mean length of hospital stay was 13.5 ± 4 days. Of 42 patients lost to follow-up at 12 months, 28 refused 12-month postsurgery consultation, 9 did not return the SF-36 questionnaire, 4 had a contralateral THR or TKR, and 1 patient died after surgery. Patients followed and those lost to follow-up at 12 months did not differ in baseline characteristics.

### Scores of satisfaction with care

Median patient satisfaction scores are in Table 1. The 2 lowest median scores were for satisfaction with *hospital environment *(score = 67) and *information *dimensions (score = 65). The highest median score was for satisfaction with *medical care *(score = 75). THR and TKR patients did not differ in satisfaction scores.

Satisfaction with care did not differ by sex. Mean scores for men and women for a *dmission *were 71 (SD = 17) and 70 (SD = 18), p = 0.86; *medical care *72 (SD = 17) and 75 (SD = 18), p = 0.41; *nursing and daily care *71 (SD = 16) and 71 (SD = 17), p = 0.96; *hospital environment *66 (SD = 17) and 69 (SD = 16), p = 0.15; and *information *69 (SD = 17) and 67 (SD = 48), p = 0.51, respectively.

### HRQoL scores over time

The mean scores at baseline and at 1, 6 and 12 months after surgery for SF-36 and clinical parameters are in Table 2. Mental health, vitality and general health scores were increased from baseline and reached a plateau at 1 month. Except for physical functioning and emotional role scores, the SF-36 score differences between satisfied and less-satisfied patients exceeded the MCID of 5 points. Physical functioning, physical role, bodily pain, social functioning and emotional role scores increased until 6 months and reached a plateau at 6 months.

**Table 2:** Mean Scores of SF36 dimensions and clinical parameters before and after total hip or knee replacement surgery

		After surgery			
		
	Before surgery mean (SD)	1 mo mean (SD)	6 mo mean (SD)	mean (SD)	p*
**SF-36**					
**Physical functioning**	36.1 (22)	39.2 (24)	56.6 (24)	59.1 (24)	< .001
**Physical role**	20.6 (32)	10.9 (24)	43.2 (42)	49.9 (42)	< .001
**Bodily pain**	33.6 (16)	53.1 (22)	59.9 (23)	61.0 (23)	< .001
**Mental health**	54.5 (19)	64.1 (20)	64.8 (20)	64.7 (19)	< .001
**Emotional role**	32.1 (41)	22.8 (38)	48.9 (46)	54.2 (44)	< .001
**Social functioning**	61.7 (23)	67.1 (25)	74.6 (23)	74.8 (23)	< .001
**Vitality**	38.8 (16)	48.5 18)	51.7 (18)	52.2 (19)	< .001
**General health**	54.7 (18)	62.6 (18)	60.9 (19)	60.6 (19)	< .001
**CLINICAL PARAMETERS**					
**Pain level (VAS, 0-100 mm)**	64.5 (17)	26.7 (20)	15.4 (17)	12.9 (16)	< .001
**Harris score (0-100)**	40.8 (13)		79.0 (11)	81.6 (13)	< .001
ISK** disease score (0-200)	100.0 (26)		165.9 (28)	173.0 (21)	< .001
**Walking distance (m)**	469 (342)		1908 (1752)	2021 (1730)	< .001

VAS = visual analog scale; ISK = Index of Severity for Knee

* ANOVA with repeated measures

### Satisfaction with care and postoperative HRQoL

Because satisfaction dimension scores were correlated, each dimension score was treated in separate models. To save space, variables significantly associated with SF-36 scores on bivariate analysis are presented for each model (in footnotes in table) [see Additional file 1]. After adjustment for preoperative SF-36 scores, age, sex, site of joint replacement, centre, and variables selected by bivariate analysis, satisfied patients had higher postoperative SF-36 scores than less-satisfied patients up to 1 year after surgery. Satisfaction with *medical care *predicted a high postoperative score for all SF-36 dimensions (p < 0.0001 to 0.03), except for physical role (p = 0.31). Satisfaction with *admission *was associated with 6 of 8 dimensions: satisfied patients showed high postoperative physical functioning, bodily pain, mental health, social functioning, vitality and general health (p = 0.0003 to 0.01). Satisfaction with *nursing and daily care *predicted 5 of 8 generic dimensions: satisfaction was associated with better bodily pain, mental health, social functioning, vitality and general health (p = 0.0004 to 0.05). Satisfaction with *hospital environment *explained a high SF-36 score for 3 variables: bodily pain (p = 0.05), vitality (p = 0.001) and general health (p = 0.0001). Finally, satisfaction with the *information *dimension was associated with general health score (p = 0.002).

Other determinants of postoperative SF-36 scores were preoperative SF-36 score, sex, age and site of joint replacement. A high preoperative SF-36 score predicted a high postoperative score in all SF-36 dimensions (p < 0.001) at the 3 post-discharge times. Whatever their preoperative score, men had higher postoperative SF-36 scores than did women for physical functioning (p = 0.003 to 0.03), bodily pain (p = 0.002 to 0.04), mental health (p = 0.007 to 0.04), vitality (p= 0.002 to 0.009) and general health (p= 0.01 to 0.02). At 12 months, for physical functioning, the mean scores were 59 (SD = 3) for men and 53 (SD = 3) for women; for bodily pain, 62 (SD = 3) and 54 (SD = 3); for mental health, 68 (SD = 2) and 62 (SD = 2); for vitality, 54 (SD = 2) and 49 (SD = 2); and for general health, 67 (SD = 2) and 61 (SD = 2). Despite similar preoperative SF-36 scores, patients with THR had better postoperative scores than those with TKR, for bodily pain (p = 0.01 to 0.02) and mental health (p = 0.01 to 0.04), whatever the time of measurement after discharge.

## Discussion

Measuring patient satisfaction with health care is an established indicator of quality of care largely used by hospital insurance companies and health policy makers to monitor quality of services. The use of this measure as a benchmark contributes to improved quality of care.

This study documents strong associations between inpatient satisfaction with care and 1-year HRQoL after surgery for bodily pain, mental health, social functioning, vitality and general health dimensions of the SF-36, whatever the preoperative HRQoL. These components predicting HRQoL suggest that actions to improve and optimize HRQoL could focus on these hospitalization features. Indeed, good communication by admission staff, physicians and nursing staff should be viewed as a core clinical skill.

As with other studies [6,29], we also showed that all scores for HRQoL significantly increased after surgery, which confirms the positive effect of THR/TKR on self-perceived health status.

The association between these satisfaction dimensions and HRQoL one year after surgery suggests that health care professional support (real or perceived) is an important determinant of HRQoL after THR or TKR, but a causal relation is still difficult to establish. Some studies of older adults have shown that personality traits such as neuroticism, conscientiousness, extraversion, openness to experience, and agreeableness could influence HRQoL [30,31], and could, although no study showed it, disturb the relation between the satisfaction with care and HRQoL after the surgery.

Patients can also develop coping strategies [32]. Further research should investigate the contribution of such coping strategies on HRQoL evolution and whether they are independent or effect modifiers.

In accordance with some studies, our study revealed other factors associated with postoperative HRQoL. Patients with good self-perceived health status have shown high postoperative HRQoL [33], but patients reporting poor preoperative HRQoL showed no improvement in HRQoL after surgery (i.e., no regression-to-the-mean phenomenon). As well, men have shown higher HRQoL after discharge than women in five HRQoL dimensions [4,11]. Many studies have shown disparities in health status between the sexes [34-37]. Men seem to benefit more from the intervention than do women, despite the high mortality rates for men. These differences can be explained largely by sex-specific variations in health behavior, acquired risk factors, and psychological and socioeconomic variables [35]. HRQoL results have invariably confirmed women to have lower HRQoL scores than men [38-40]. Patients with THR showed higher HRQoL than did patients with TKR [4,6,41,42]. Although patients undergoing THR and TKR are hospitalized in the same surgical centers and are treated by the same medical and ancillary staff in each setting, patients with THR recover faster and show a better HRQoL than those with TKR [4,41]. Our results also found age not associated with postoperative HRQoL, which confirms previous findings [43]. Contrary to other studies showing that obesity and comorbities negatively influence SF-36 or WOMAC scores [4,5,9,44,45], we did not observe any effect of these parameters on postoperative SF-36 scores. This disagreement could be explained by a difference in sample characteristics. Our study included fewer patients with severe or morbid obesity than did other studies: 75% of patients had a BMI ≤ 30, whereas in the most recent study [45], more than 50% of patients had a BMI > 30. Consequently, we cannot exclude an effect of higher BMI on our results. As well, the lack of association with comorbidity could be explained by the low frequency of patients with comorbidities in our sample: almost 90% of our patients declared 0 or 1 comorbidities at inclusion.

This study has some limitations that could restrict the generalization of our findings. First, the mean length of hospital stay (13.5 days) was long as compared with practices reported in other countries. A short hospital stay is likely to influence the level of inpatient satisfaction, as was recently suggested [44]. In our study, the length-of-stay distribution was rather narrow (see table 1), so if length of stay affects HRQoL, this impact was of similar magnitude for all patients, which limits a potential impact of variability and subsequent association of satisfaction with care and other parameters and HRQoL. Second, the patients were recruited from several hospitals in one French region, so generalizability of results remains uncertain. Finally, if THR and TKR can improve HRQoL, the benefits may be time limited or dependent [46]. We studied the impact of patient satisfaction with care on HRQoL one year after surgery; therefore, environmental factors and treating comorbidities and pain in locations other than those of the arthroplasty could have mid- and long-term effects on QoL of patients with THR or TKR and may modify the relation between immediate postoperative satisfaction and HRQoL after surgery.

## Conclusion

Patients with OA undergoing THR or TKR who were satisfied with the admission process and medical and nursing care related to their surgery were more likely to have better scores on HRQoL dimensions up to 1 year after surgery, which reveals new factors to improve OA patients' HRQoL after such surgery. This finding suggests the use of inpatient satisfaction measures beyond judging the quality of hospital care. These satisfaction measures will bring new insights into clinical practice because, besides being an indicator of quality of care, immediate postoperative satisfaction with care seems to be a good predictor of self-perceived health status after surgery. To improve HRQoL for patients, the hospital could focus on the efficiency of the admission procedure (improving the attention of admitting staff to patient needs) and medical and nursing care (attention, skill, information given, and coordination of care). Finally, the predictive value of satisfaction with care, measured shortly after hospital discharge, on medium-term HRQoL of OA patients after THR and TKR represents a pertinent indicator that is quickly accessible to clinicians. Whether this finding is true in the long term after THR/TKR and for other chronic diseases and other hospital departments remains for further investigation.

## List of abbreviations

HRQoL: Health-Related Quality Of Life; OA: osteoarthritis; THR: total hip or knee replacement; TKR: total knee replacement; SF-36: Medical Outcomes Study 36-item Short Form; PJHQ: Patient Judgements of Hospital Quality.

## Competing interests

The authors declare that they have no competing interests.

## Authors' contributions

Each author has made substantive intellectual contributions to this multicentre study:

CB: statistical analysis, interpretation of data, writing manuscript; ACR: statistical analysis, interpretation of data, manuscript revision; GO: acquisition of data, manuscript revision; DM: design of study, acquisition of data, manuscript revision; CC: acquisition of data, manuscript revision; FG: conception of study, interpretation of data, manuscript revision.

The authors have given final approval of the version to be published.

## Pre-publication history

The pre-publication history for this paper can be accessed here:

http://www.biomedcentral.com/1471-2474/10/150/prepub

## Supplementary Material

Additional file 1 **Comparison of postoperative health-related quality of life (SF-36) dimensions between patients satisfied (Score > 70) and those less satisfied (score ≤ 70) with care**. table presents the results of multivariate analysis. Click here for file
