# Supplementary material for: Satisfaction with care after total hip or knee replacement predicts self-perceived health status after surgery
Source: BMC Musculoskelet Disord. 2009 Dec 3;10:150. doi: 10.1186/1471-2474-10-150 (PMC2795735; doi:10.1186/1471-2474-10-150)
Supplement: Additional file 1 — Comparison of postoperative health-related quality of life (SF-36) dimensions between patients satisfied (Score > 70) and those less satisfied (score ≤ 70) with care. table presents the results of multivariate analysis. [file 1471-2474-10-150-S1.DOC]

**Comparison of postoperative health-related quality of life (SF-36) dimensions between patients satisfied (Score > 70) and those less satisfied (score < 70) with care**

|  | **Physical functioning** (1)* | | | | |  | **Physical role** (2) | | | |  | **Bodily pain** (3) | | | |  | **Mental health** (4) | | | |
| --- | --- | --- | --- | --- | --- | --- | --- | --- | --- | --- | --- | --- | --- | --- | --- | --- | --- | --- | --- | --- |
|  | mo 1 | mo 6 | | mo 12 | p |  | mo 1 | mo 6 | mo 12 | p |  | mo 1 | mo 6 | mo 12 | p |  | mo 1 | mo 6 | mo 12 | p |
|  | m [_95_CI] | m [_95_CI] | | m [_95_CI] |  |  | m [_95_CI] | m [_95_CI] | m [_95_CI] |  |  | m [_95_CI] | m [_95_CI] | m [_95_CI] |  |  | m [_95_CI] | m [_95_CI] | m [_95_CI] |  |
| **Admission** | |  | |  | 0.01 |  |  |  |  | 0.61 |  |  |  |  | 0.002 |  |  |  |  | 0.0006 |
| Satisfied | 44 [36-52] | 66 [58-74] | | 67 [59-75] |  | | 12 [2-22] | 49 [37-61] | 57 [45-69] |  | | 56 [50-62] | 65 [59-71] | 66 [60-72] |  | | 70 [66-74] | 69 [65-73] | 68 [64-72] |  |
| Less satisfied | 37 [29-45] | 62 [52-72] | | 62 [54-70] |  |  | 19 [9-29] | 50 [40-60] | 54 [44-64] |  |  | 49 [43-55] | 57 [51-63] | 57 [53-61] |  |  | 61 [57-65] | 62 [58-66] | 63 [59-67] |  |
| **Medical care** | |  | |  | 0.02 |  |  |  |  | 0.31 |  |  |  |  | 0.0007 |  |  |  |  | 0.0001 |
| Satisfied | 44 [36-52] | 63 [55-71] | | 66 [58-74] |  | | 18 [8-28] | 50 [40-60] | 58 [48-68] |  | | 58 [52-64] | 64 [58-70] | 65 [59-71] |  | | 70 [66-74] | 68 [64-72] | 67 [61-73] |  |
| Less satisfied | 40 [32-48] | 58 [50-66] | | 59 [51-67] |  |  | 16 [6-26] | 47 [35-59] | 52 [40-64] |  |  | 47 [41-53] | 54 [48-60] | 57 [53-63] |  |  | 58 [54-62] | 61 [57-65] | 61 [57-65] |  |
| **Nursing and daily care** | | |  |  | 0.13 |  |  |  |  | 0.90 |  |  |  |  | 0.05 |  |  |  |  | 0.0008 |
| Satisfied | 44 [36-50] | 62 [54-70] | | 65 [57-73] |  | | 17 [7-27] | 47 [37-57] | 57 [47-67] |  | | 57 [51-63] | 63 [57-69] | 64 [58-70] |  | | 70 [66-74] | 69 [65-73] | 68 [64-72] |  |
| Less satisfied | 40 [32-48] | 60 [52-68] | | 61[53-69] |  |  | 15 [5-25] | 51 [41-61] | 54 [44-64] |  |  | 50 [46-54] | 58 [52-64] | 60 [54-66] |  |  | 61 [57-65] | 62 [58-66] | 63 [59-67] |  |
| **Hospital environment** | | | |  | 0.18 |  |  |  |  | 0.88 |  |  |  |  | 0.05 |  |  |  |  | 0.15 |
| Satisfied | 44 [36-52] | 63 [55-71] | | 66 [58-74] |  | | 17 [5-29] | 51 [39-63] | 55 [43-67] |  | | 57 [51-63] | 65 [59-71] | 64 [58-70] |  | | 70 [66-74] | 66 [62-70] | 66 [62-70] |  |
| Less satisfied | 41 [35-47] | 61 [53-69] | | 62 [56-68] |  |  | 13 [3-23] | 50 [40-60] | 57 [47-67] |  |  | 50 [44-56] | 59 [53-65] | 61 [55-67] |  |  | 62 [58-66] | 65 [62-70] | 65 [61-69] |  |
| **Information** | |  | |  | 0.95 |  |  |  |  | 0.15 |  |  |  |  | 0.76 |  |  |  |  | 0.07 |
| Satisfied | 40 [32-48] | 59 [51-67] | | 64 [56-72] |  | | 11 [1-21] | 44 [30-58] | 46 [34-48] |  | | 53 [45-61] | 59 [51-67] | 62 [54-70] |  | | 68 [62-74] | 66 [60-72] | 65 [59-71] |  |
| Less satisfied | 42 [34-50] | 60 [52-68] | | 61 [53-69] |  |  | 15 [3-27] | 51 [39-63] | 55 [43-67] |  |  | 51 [43-59] | 59 [51-67] | 60 [52-68] |  |  | 61 [55-67] | 63 [57-69] | 61 [55-67] |  |
| * Dimension (model) | | | | | | | | | | | | | | | | | | | | |
| m [_95_CI]: adjusted mean [95% Confidence Interval] | | | | | | | | | | | | | | | | | | | | |
| P: test of HRQoL scores between satisfied and less unsatisfied patients, linear model with repeated measures | | | | | | | | | | | | | | | | | | | | |
| Models adjusted on preoperative quality of life QoL (models 1 to 4), age (1-4), sex (1-4), site of joint replacement (1-4), centre (1-4), years of schooling (models 1 and 2), preoperative pain level by visual analog scale VAS (models 1, 3 and 4), preoperative walking distance (model 3), Charlson comorbidity index (model 1), length-of-stay (model 3) | | | | | | | | | | | | | | | | | | | | |

**Comparison of postoperative health-related quality of life (SF-36) dimensions between patients satisfied (Score > 70) and those less satisfied (score < 70) with care (Continued)**

|  | | **Emotional role** (5)* | | | |  | **Social functioning** (6) | | | |  | **Vitality** (7) | | | |  | **General health** (8) | | | |
| --- | --- | --- | --- | --- | --- | --- | --- | --- | --- | --- | --- | --- | --- | --- | --- | --- | --- | --- | --- | --- |
|  | | mo 1 | mo 6 | mo 12 | p |  | mo 1 | mo 6 | mo 12 | p |  | mo 1 | mo 6 | mo 12 | p |  | mo 1 | mo 6 | mo 12 | p |
|  | | m [_95_CI] | m [_95_CI] | m [_95_CI] |  |  | m [_95_CI] | m [_95_CI] | m [_95_CI] |  |  | m [_95_CI] | m [_95_CI] | m [_95_CI] |  |  | m [_95_CI] | m [_95_CI] | m [_95_CI] |  |
| **Admission** | | |  |  | 0.13 |  |  |  |  | 0.007 |  |  |  |  | 0.0003 |  |  |  |  | 0.001 |
| Satisfied | | 31 [21-41] | 58 [48-68] | 60 [50-70] |  |  | 71 [65-77] | 80 [74-86] | 80 [74-86] |  |  | 53 [49-57] | 56 [52-60] | 56 [52-60] |  |  | 70 [66-74] | 68 [64-72] | 67 [63-71] |  |
| Less satisfied | | 23 [11-35] | 48 [36-60] | 61 [49-73] |  |  | 66 [60-72] | 71 [65-77] | 70 [64-73] |  |  | 45 [41-49] | 49 [45-53] | 49 [45-53] |  |  | 62 [58-66] | 60 [54-66] | 60 [56-64] |  |
| **Medical care** | | |  |  | 0.03 |  |  |  |  | 0.002 |  |  |  |  | 0.0002 |  |  |  |  | <.0001 |
| Satisfied | | 28 [18-38] | 56 [44-68] | 61 [51-71] |  |  | 72 [66-78] | 77 [71-83] | 78 [72-84] |  |  | 54 [50-58] | 54 [50-58] | 55 [49-61] |  |  | 69 [65-73] | 67 [63-71] | 67 [61-73] |  |
| Less satisfied | | 23 [13-33] | 40 [30-50] | 51 [41-61] |  |  | 62 [56-68] | 68 [62-74] | 68 [62-74] |  |  | 44 [40-48] | 48 [44-52] | 48 [44-52] |  |  | 60 [56-64] | 58 [54-62] | 57 [53-61] |  |
| **Nursing and daily care** | | | |  | 0.21 |  |  |  |  | 0.03 |  |  |  |  | 0.0004 |  |  |  |  | 0.001 |
| Satisfied | 29 [19-39] | | 56 [44-68] | 62 [50-74] |  |  | 71 [65-77] | 79 [73-85] | 77 [71-83] |  |  | 54 [50-58] | 55 [51-59] | 55 [51-59] |  |  | 68 [64-72] | 67 [63-71] | 66 [62-70] |  |
| Less satisfied | 26 [16-36] | | 47 [35-59] | 56 [44-68] |  |  | 65 [59-71] | 71 [65-77] | 72 [66-78] |  |  | 44 [40-48] | 49 [41-49] | 50 [46-54] |  |  | 62 [58-66] | 59 [55-63] | 59 [55-63] |  |
| **Hospital environment** | | | |  | 0.51 |  |  |  |  | 0.46 |  |  |  |  | 0.001 |  |  |  |  | 0.0001 |
| Satisfied | | 29 [17-41] | 55 [43-67] | 60 [48-72] |  |  | 72 [66-78] | 76 [70-82] | 75 [69-81] |  |  | 54 [50-58] | 55 [51-59] | 56 [52-60] |  |  | 70 [66-74] | 67 [63-71] | 67 [63-71] |  |
| Less satisfied | | 26 [16-36] | 50 [40-60] | 58 [48-68] |  |  | 65 [59-71] | 75 [69-81] | 75 [69-81] |  |  | 45 [41-49] | 50 [46-54] | 51 [47-55] |  |  | 61 [57-65] | 60 [56-64] | 59 [55-63] |  |
| **Information** | | |  |  | 0.99 |  |  |  |  | 0.62 |  |  |  |  | 0.11 |  |  |  |  | 0.002 |
| Satisfied | | 16 [2-30] | 56 [42-68] | 53 [39-67] |  |  | 66 [58-72] | 75 [67-83] | 73 [65-81] |  |  | 51 [45-57] | 52 [46-58] | 54 [48-60] |  |  | 68 [62-74] | 67 [61-73] | 66 [60-72] |  |
| Less satisfied | | 28 [14-42] | 45 [31-59] | 52 [38-66] |  |  | 68 [60-76] | 71 [63-79] | 69 [59-79] |  |  | 47 [46-53] | 50 [44-56] | 49 [43-55] |  |  | 60 [54-66] | 59 [53-65] | 56 [50-62] |  |
| * Dimension (model) | | | | | | | | | | | | | | | | | | | | |
| m [_95_CI]: adjusted mean [95% Confidence Interval] | | | | | | | | | | | | | | | | | | | | |
| P: test of HRQoL scores between satisfied and less satisfied patients, Manova with repeated measures | | | | | | | | | | | | | | | | | | | | |
| Models adjusted on preoperative quality of life (models 5 to 8), age (5-8), sex (5-8), site of joint replacement (5-8), centre (5-8), years of schooling (models 5, 6 and 7), preoperative pain level by visual analog scale (model 6), preoperative walking distance (models 6 and 7), Charlson comorbidity index (model 5), length-of-stay (model 6), preoperative Harris score/Index of Severity for Knee score (model 6), marital status (model 5), Rehabilitation center (model 8). | | | | | | | | | | | | | | | | | | | | |
